# Supplementary material for: Interaction Modes of Microsomal Cytochrome P450s with Its Reductase and the Role of Substrate Binding
Source: Int J Mol Sci. 2020 Sep 11;21(18):6669. doi: 10.3390/ijms21186669 (PMC7555755; doi:10.3390/ijms21186669)
Supplement: Supplementary file 1 [file ijms-21-06669-s001.pdf]

Supplementary Material

Interaction Modes of Microsomal Cytochrome P450s with Its Reductase and the Role of Substrate Binding

Francisco Esteves [1,*], Philippe Urban [2], José Rueff [1], Gilles Truan [2] and Michel Kranendonk [1,*]

[1] Center for Toxicogenomics and Human Health (ToxOmics), Genetics, Oncology and Human Toxicology, NOVA Medical School/Faculty of Medical Sciences, Universidade NOVA de Lisboa, 1169-056 Lisboa, Portugal; jose.rueff@nms.unl.pt (J.R.)

[2] TBI, Université de Toulouse, CNRS, INRAE, INSA, CEDEX 04, 31077 Toulouse, France; philippe.urban@insa-toulouse.fr (P.U.); gilles.truan@insa-toulouse.fr (G.T.)

* Correspondence: francisco.esteves@nms.unl.pt (F.E.); michel.kranendonk@nms.unl.pt (M.K.)

Supplementary Tables

Supplementary Table S1. Kinetic steady-state parameters of CYP1A2, 2A6 and 3A4-activities.

CYP isoform	CPR form	k_{cat} (product formed pmol.min^{-1}.pmol CYP^{-1})	K_M (µM)
CYP1A2 (EROD)	wt	0.59 ± 0.02	0.84 ± 0.07
	P117H	0.71 ± 0.01 **	0.34 ± 0.03 ***
	G144C	0.76 ± 0.01 ***	0.42 ± 0.02 **
	G175D	0.52 ± 0.01 **	0.35 ± 0.02 ***
	N151D	0.44 ± 0.01 ***	0.23 ± 0.01 ***
CYP2A6 (C7H)	wt	1.51 ± 0.08	2.39 ± 0.41
	P117H	1.43 ± 0.07	1.66 ± 0.29
	G144C	1.26 ± 0.07 *	2.89 ± 0.46
	G175D	2.03 ± 0.10 **	3.70 ± 0.73
	N151D	1.32 ± 0.04 *	1.77 ± 0.20
CYP3A4 (DBODF)	wt	3.35 ± 0.08	0.74 ± 0.07
	P117H	2.93 ± 0.15 *	0.96 ± 0.16
	G144C	2.81 ± 0.07 **	0.68 ± 0.06
	G175D	4.08 ± 0.10 **	0.96 ± 0.08 *
	N151D	5.38 ± 0.21 ***	1.92 ± 0.21 **

Kinetic parameters values of the CPR_{mut}/CYP were compared with the ones of the CPR_{wt}/CYP applying the unpaired *t* test (technical replicates N=3). Control experiments with CPR_{null}/CYP membrane fractions did not show any detectable activities with the respective substrates. Color code is the same as of Table 1. Ethoxy-resorufin O-deethylation (EROD); Coumarin 7-hydroxylation (C7H); Dibenzylfluorescein O-debenzylation (DBODF).

*** $P < 0.0005$; ** $P < 0.005$; * $P < 0.05$.

Supplementary Table S2. Kinetic steady-state parameters of CYP1A2-activities with CEC and DBF.

Reaction	CPR form	k_{cat} (product formed pmol.min^{-1}.pmol CYP^{-1})	K_M (μM)
**CECOD**	**wt**	0.52 ± 0.03	10.89 ± 1.47
	**P117H**	0.49 ± 0.02	3.57 ± 0.55 **
	**G144C**	0.57 ± 0.02	5.71 ± 0.57 **
	**G175D**	0.42 ± 0.01 *	10.63 ± 0.67
	**N151D**	0.29 ± 0.01 ***	3.92 ± 0.44 **
**DBODF**	**wt**	1.85 ± 0.18	17.59 ± 3.58
	**P117H**	1.71 ± 0.14	17.85 ± 3.20
	**G144C**	1.82 ± 0.10	22.02 ± 2.50
	**G175D**	3.38 ± 0.19 ***	30.60 ± 3.10 *
	**N151D**	1.73 ± 0.20	17.63 ± 4.46

Kinetic parameters values of the CPR_{mut}/CYP1A2 were compared with the ones of the CPR_{wt}/CYP1A2 applying the unpaired *t* test (technical replicates N=3). Control experiments with CPR_{null}/CYP1A2 membrane fraction did not show any detectable activities with the respective substrates. Color code is the same as of Table 1. 3-cyano-7-ethoxycoumarin O-dealkylation (CECOD); Dibenzylfluorescein O-debenzylation (DBODF).

*** $P < 0.0005$; ** $P < 0.005$; * $P < 0.05$.

Supplementary Table S3. Summary of determinant CPR residues directly involved in hydrogen bonds/salt bridges on both FD:cyt *c* and FD:CYP binding sites.

FD residues involved in interaction with		
cyt *c*		CYP
Cluster I	Cluster II	
Q90	Q90	D116
E95	**E145**	E118
E96	**D150**	E119
**D211**	E182	**E145**
D212	H183	D147
E216		**D150**
E217		**D211**

Common FD:cyt *c* (two clusters) [17,25,44,45,48] and FD:CYP [14,24,25,43,45,46,49] complexes interfaces are referred in bold blue. Residue numbering is according to human CPR NCBI consensus protein sequence NP_000932.3.

Supplementary Figures

Supplementary Figure S1

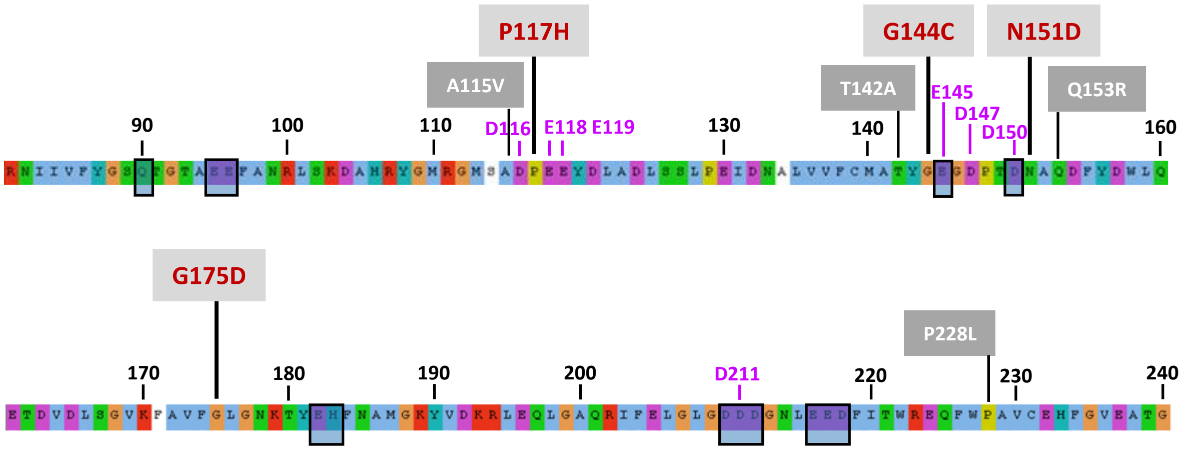

Supplementary Figure S1. Protein sequence of the human CPR FD. Resource (UniProt) (www.uniprot.org), using the Clustal X color scheme for amino acid alignments. Residue numbering (in black) is shown according to the human CPR NCBI consensus protein sequence NP_000932.3. Mutations studied are indicated with red numbering within grey boxes, on top of the sequence, residues found in natural FD variants (with CYP isoform specific effects) are white numbered within dark grey boxes, acidic amino acid stretches involved in CPR:CYP interactions in purple, and residues potentially located on the CPR:cyt *c* complex interface in black lined boxes.

Supplementary Figure S2

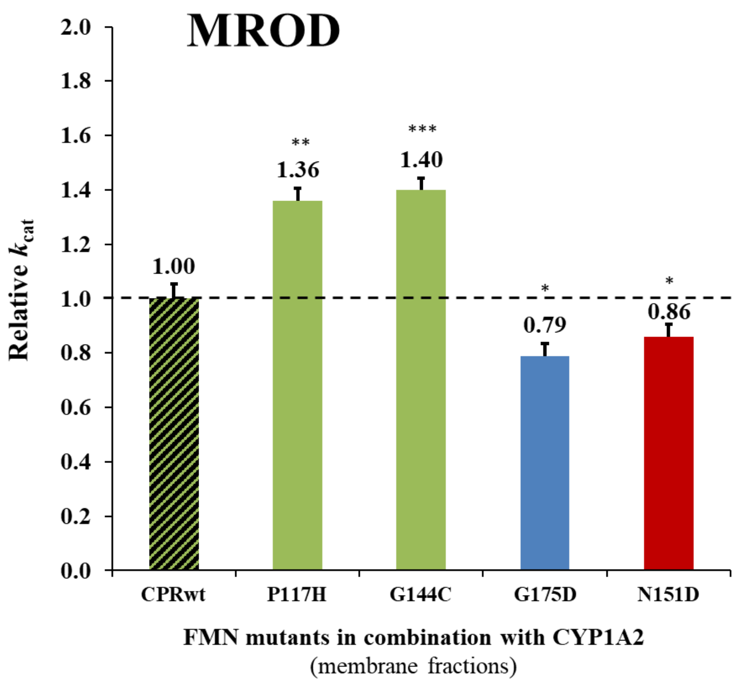

Supplementary Figure S2. Relative turnover rates (k_{cat}) (x fold) of the CPR_{mut}:CYP1A2 MROD activities (using 0-2 µM MthR), normalized by the k_{cat} demonstrated by CPR_{wt}/CYP (determined in membrane fractions) (technical replicates N=3). FD wildtype (CPR_{wt}:CYP, black stripes). FD mutants previously demonstrated to support a gain in CYP1A2-mediated EROD- (P117H and G144C, green), CYP2A6-mediated C7H- (G175D, blue), and CYP3A4-mediated DBODF-activity (N151D, red). Values depicted for CPR_{P117H}/CYP1A2 and CPR_{G144C}/CYP1A2 were reported in our previous study [16]. k_{cat} values of the CPR_{mut}/CYP1A2 were compared with the ones of the CPR_{wt}/CYP1A2 applying the unpaired *t* test.

*** $P < 0.0005$; ** $P < 0.005$; * $P < 0.05$.

Supplementary Figure S3

A)

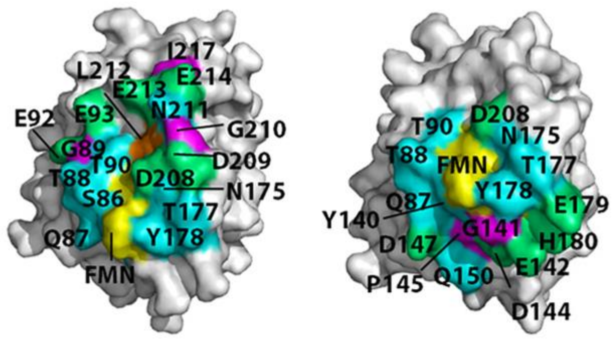

B)

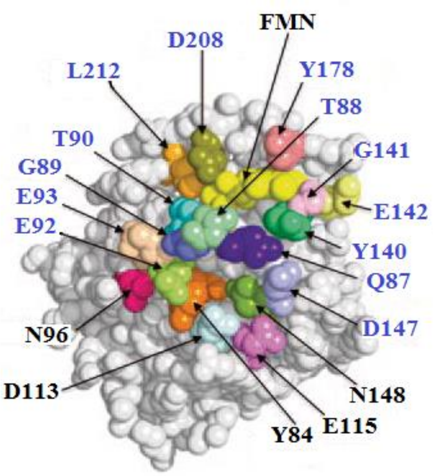

Supplementary Figure S3. Structural models of the FD binding site of rat CPR. **A**, representative of the two FD interface clusters of the lowest energy complex structures generated by high ambiguity driven biomolecular docking (HADDOCK) (PDB 1AMO) (adapted from Huang et al., 2015) [44]. **B**, model of the interface residues of the FD, described to be in direct contact with CYP2B4 proximal site in a FD:CYP2B4 model generated using mutagenesis constrains (PDB 1JA1) (adapted from Waskell and Kim, 2015; Nicolo et al., 2010) [5,47]. Interface residues involved in protein:protein contacts in both models are highlighted and coloured. FMN cofactor is coloured in yellow. Residues are identified with the single-letter amino acid code. In **B**, residues involved in interactions with CYP2B4 and cyt *c* are indicated in blue.

Supplementary Figure S4

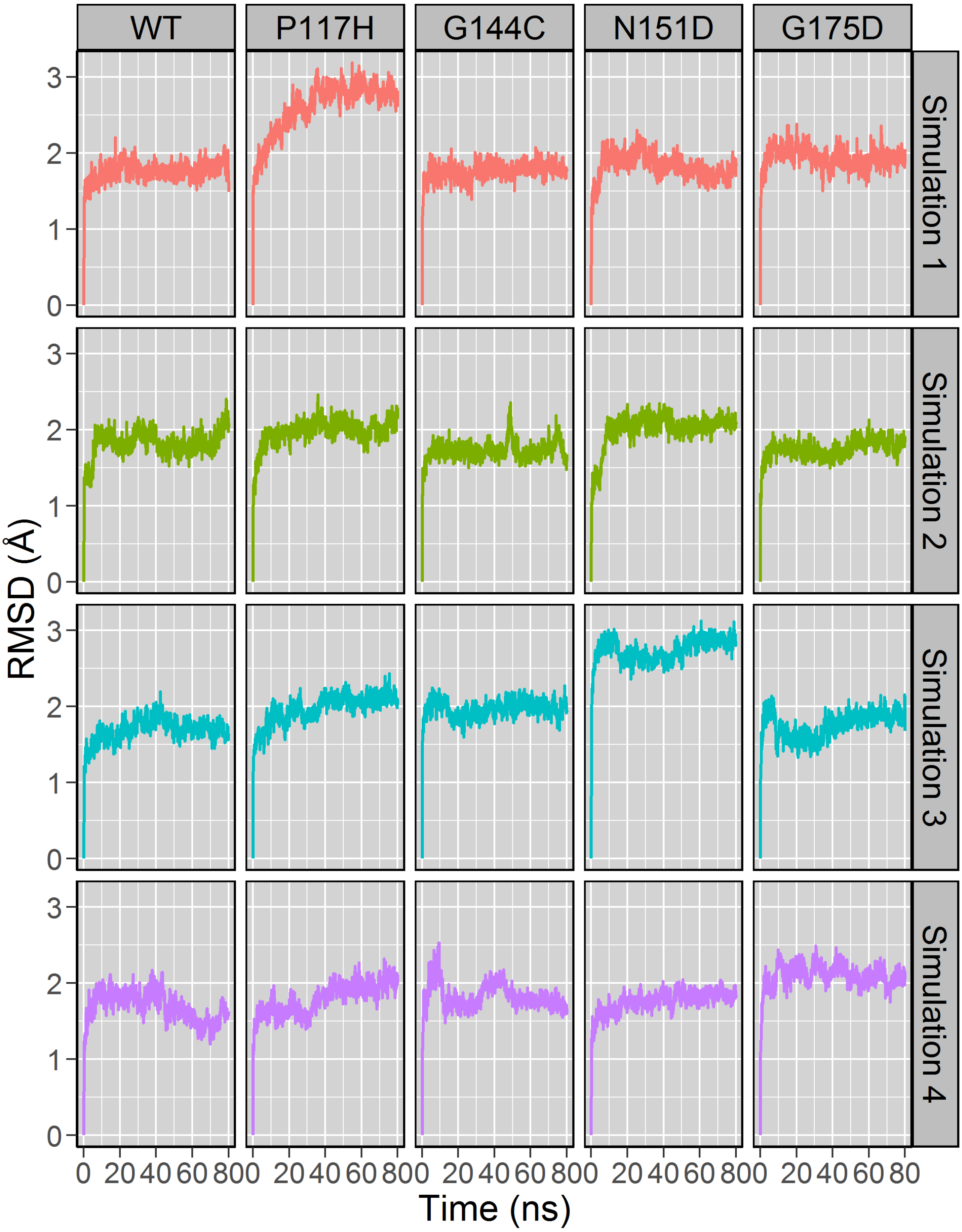

Supplementary Figure S4. RMSD of the 80 ns simulations. Each snapshot was structurally aligned onto the first snapshot. RMSD was calculated for each snapshot using the Bio3D suite for R and plotted against the snapshots (800 snapshots).

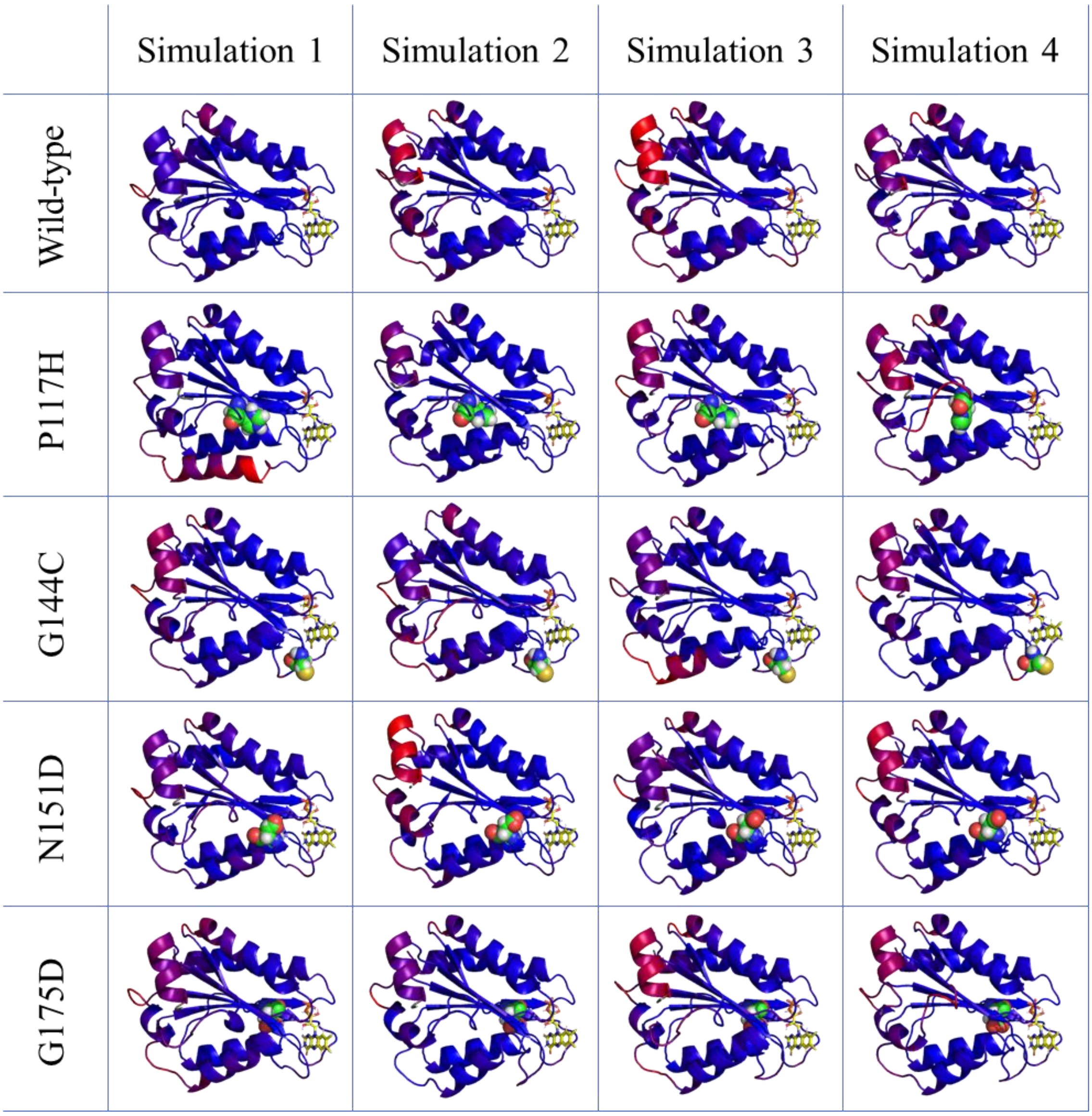

Supplementary Figure S5. Comparison of the deviations between each average structure obtained from simulation and the FMN domain from the crystal structure of human CPR (PDB 5FA6). Each structure was aligned onto the FMN domain and RMSDs calculated between α-carbons for all 166 residues. Average structures were colored by the RMSD values using the colorbyRMSD function of Pymol, blue and red representing low and high RMSDs, respectively. Mutations are represented as spheres.

Supplementary Figure S6

A

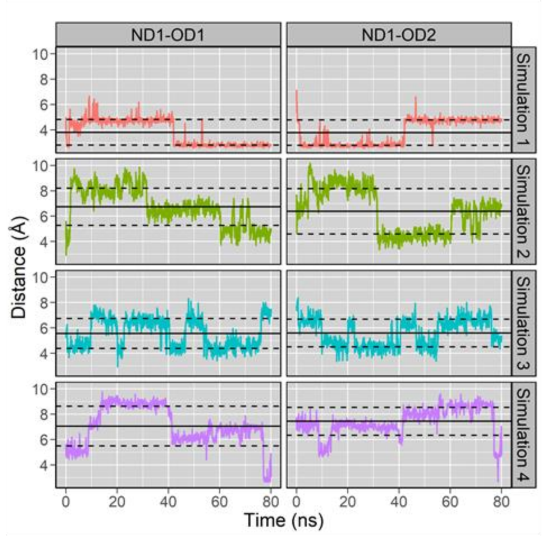

B

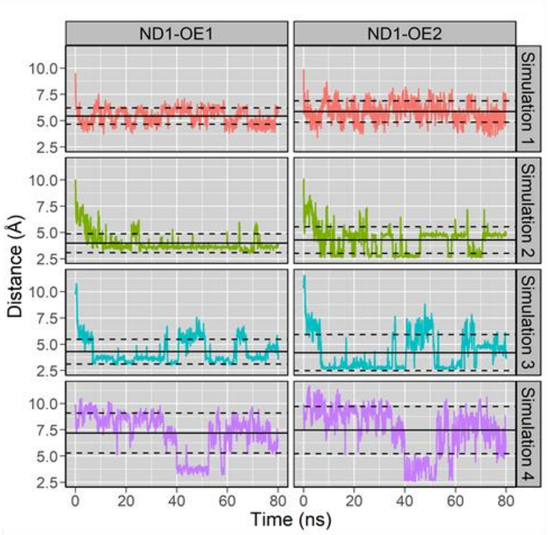

C

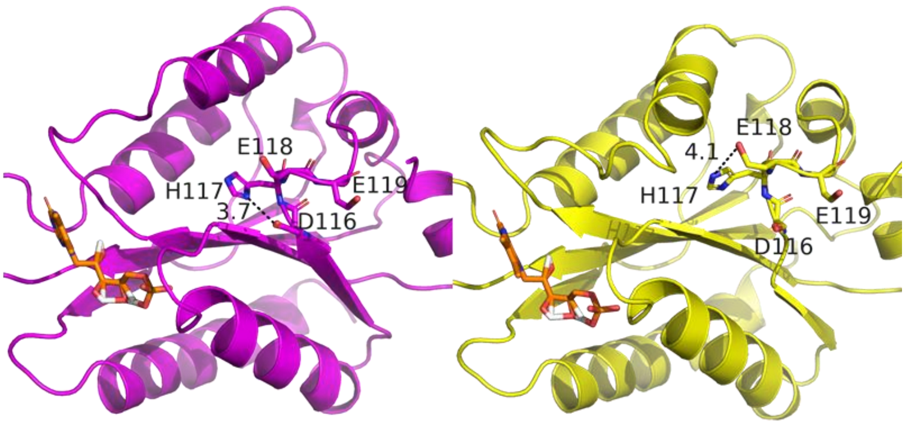

Supplementary Figure S6. Hydrogen bonds between H117 and D116 or E118 in the P117H mutant. Panel **A** and **B**. Distances between the ND1 atom and either the OD1 and OD2 (D116, Panel **A**) or OE1 and OE2 (E118, Panel **B**) were calculated on the 800 snapshot structures generated with four independent molecular dynamics simulations (Simulation 1 to 4) using the Bio3D suite in R. The straight horizontal line represents the average distance, the dashed lines represent the average distance ± one standard deviation. Panel **C** represents two P117H average structures from two simulations (magenta, simulation 1; yellow, simulation 2). The hydrogen bonds of P117H with D116 (simulation 1) and E118 (simulation 2) are represented by dashed lines along with the distances calculated on the average structures.

Supplementary Figure S7

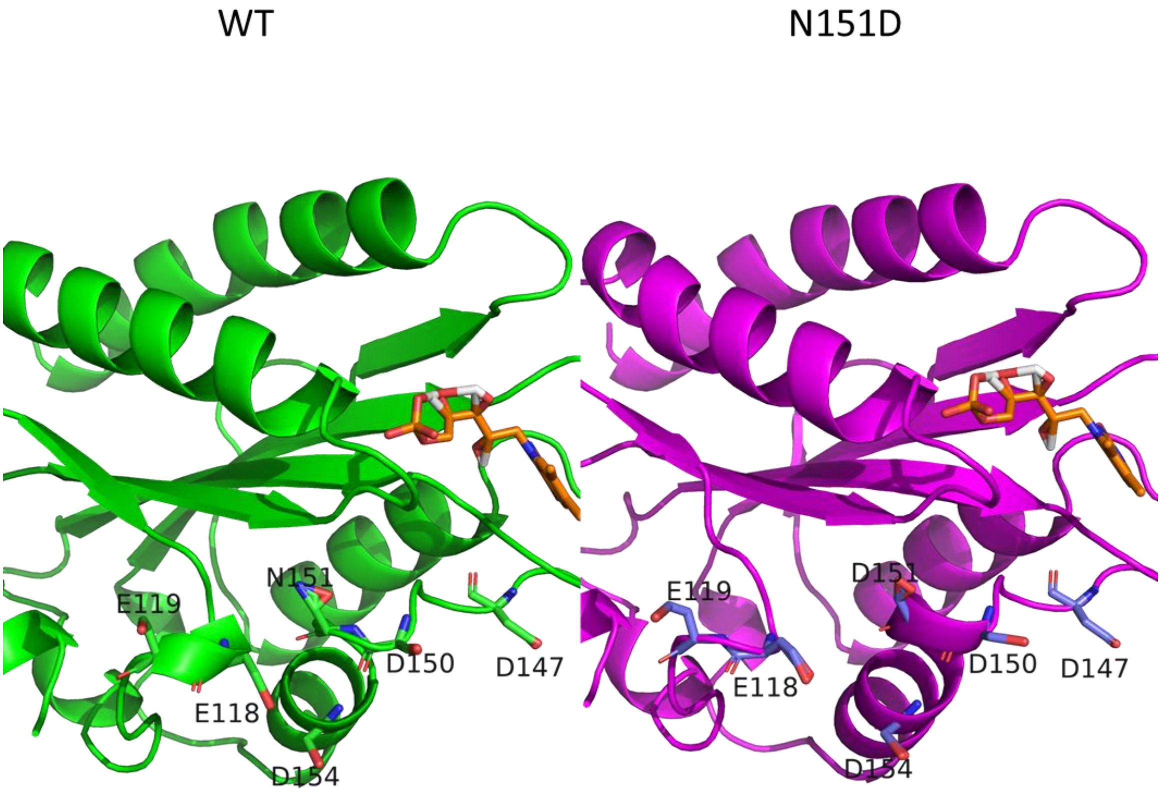

Supplementary Figure S7. Acidic residues surrounding the aspartate 151 in the N151 D mutant. The wild-type (green, simulation 1) and N151D average structures (magenta, simulation 1) are represented.

Supplementary Figure S8

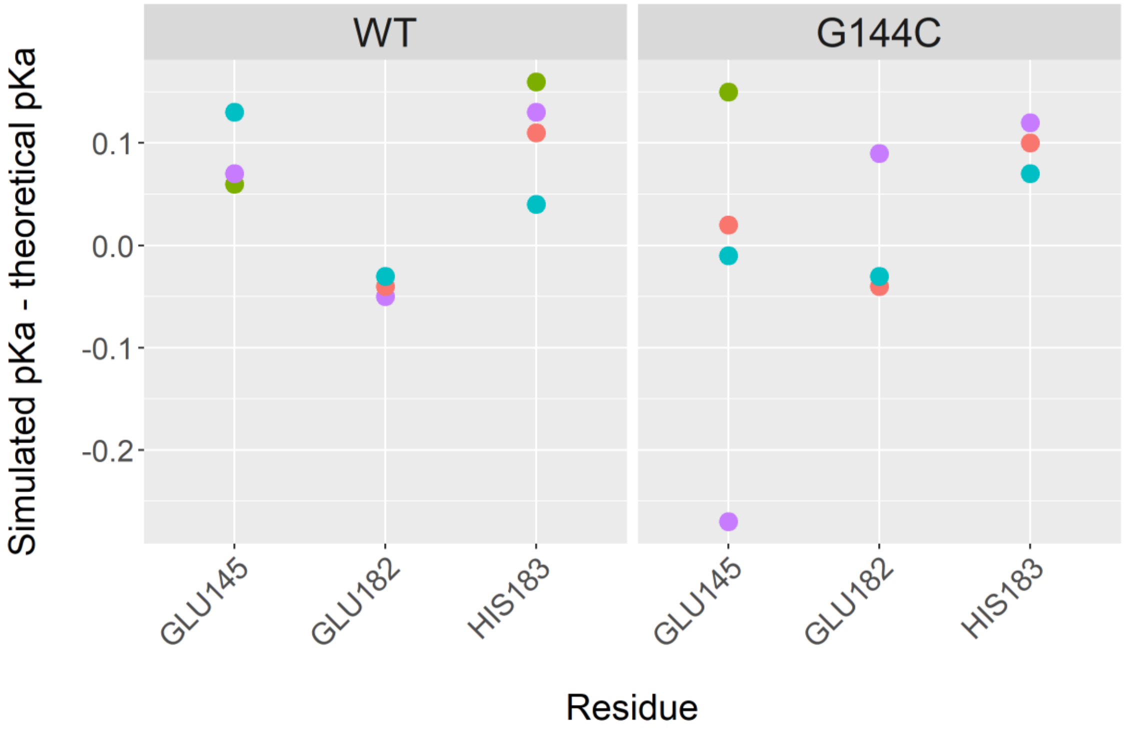

Supplementary Figure S8. Variation of pKa of the surrounding charged residues for G144C mutant. pKa values were calculated on the average structures generated with four independent molecular dynamics simulations for wild-type (WT) or G144C (G144C) with the propka options of the pdb2pqr software, using a pH=7.5 value and the CHARMM force field. Theoretical pKa values of glutamate (4.5) or histidine (6.5) were then subtracted from the simulated ones.

Supplementary Figure S9

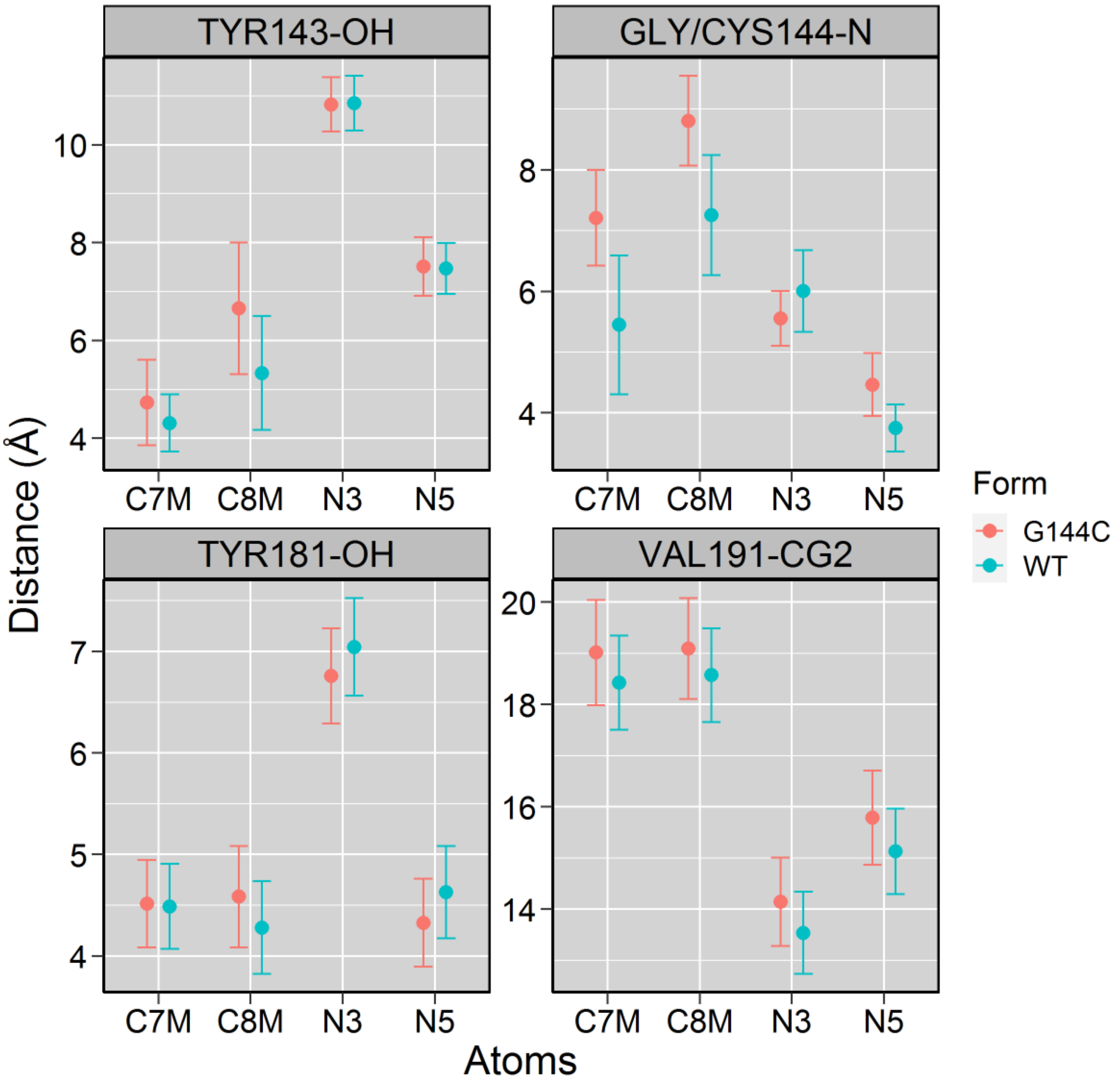

Supplementary Figure S9. Distances between the OH atom of Y143 or Y181, N atom of C144 or G144, CG2 atom of V191 and either the C7M, C8M, N3 or N5 atoms of FMN in WT or G144C mutant. Distances were calculated on the 800 snapshot structures generated with four independent molecular dynamics simulations using the Bio3D suite in R. Dots represents the average distance ± one standard deviation.

Supplementary Figure S10

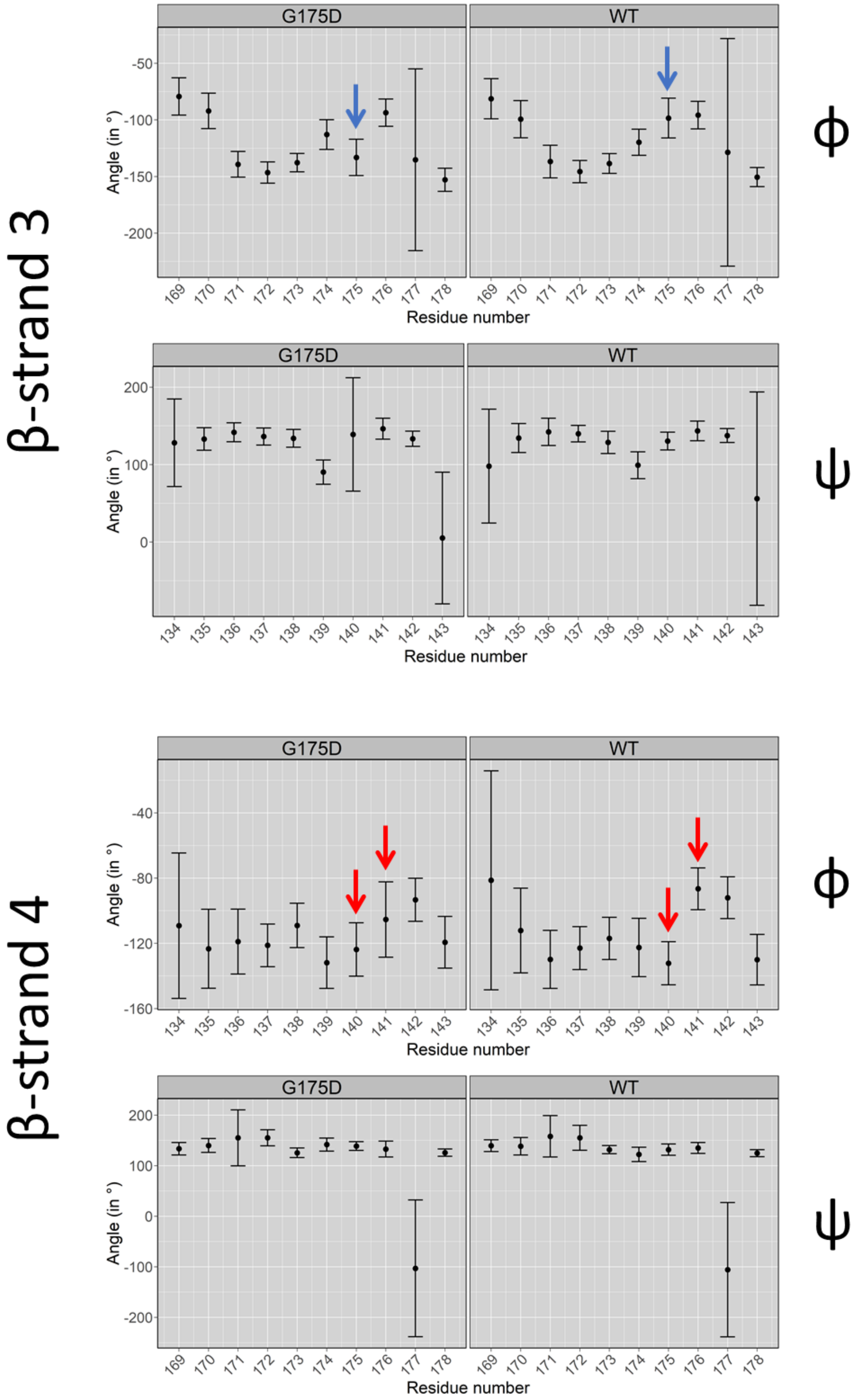

Supplementary Figure S10. Analyses of the various φ and ψ angles for β-strands 3 and 4. Angles were calculated on the 800 snapshot structures generated with four independent molecular dynamics simulations using the Bio3D suite in R and then averaged ± one standard deviation.

Supplementary Figure S11

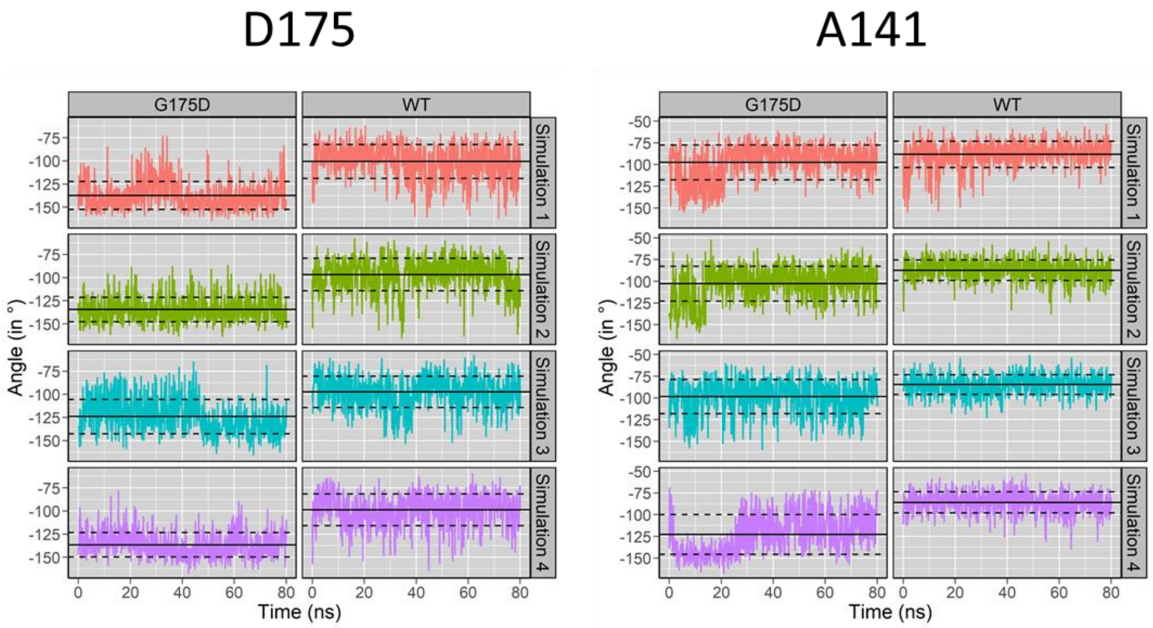

Supplementary Figure S11. Analysis of the φ angles along the four simulations for residues 175 and 141 in the WT and G175 FD mutant. The horizontal plain line represents the average φ angle and the dashed lines the average angle ± one standard deviation.

Supplementary Figure S12

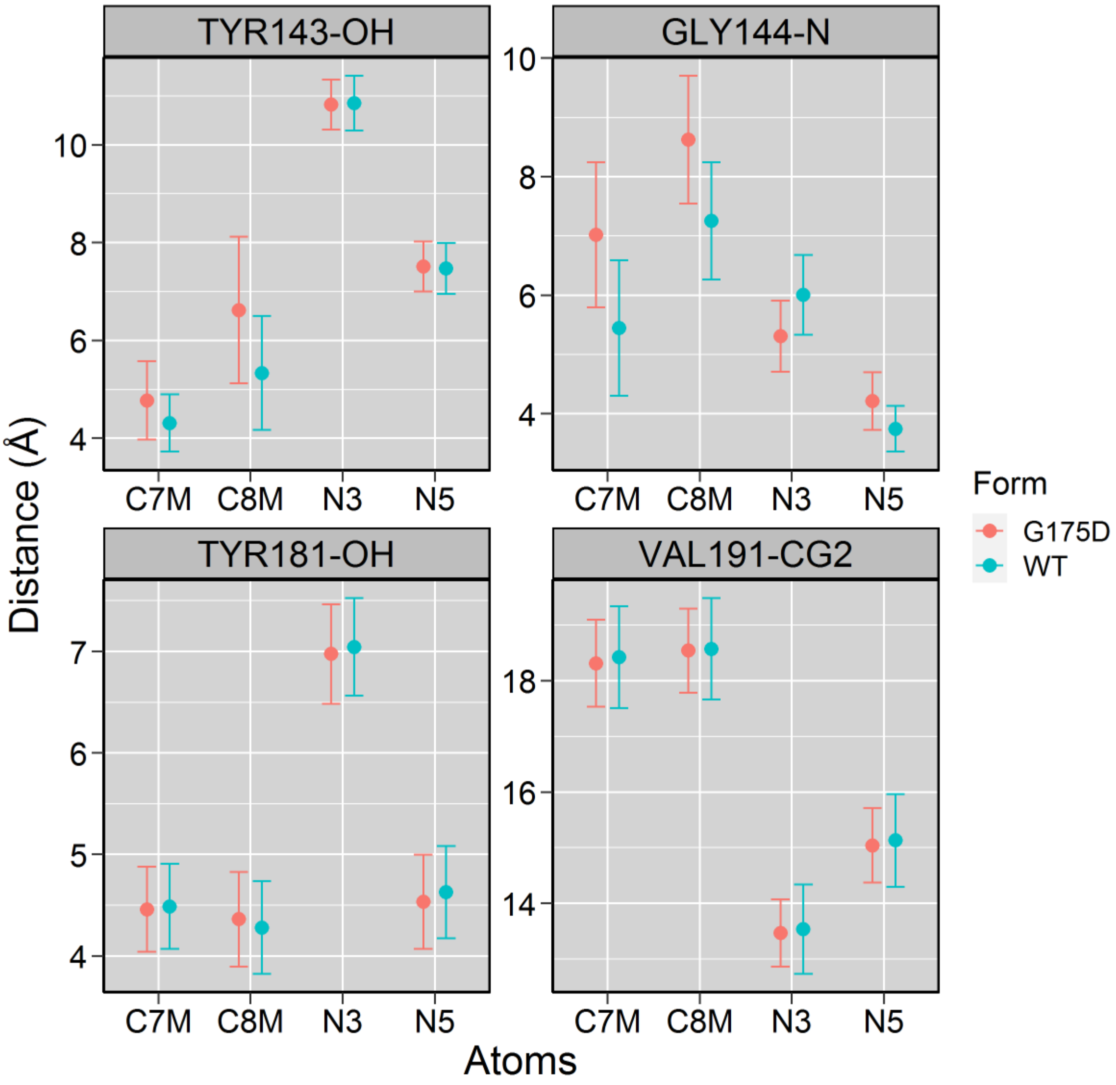

Supplementary Figure S12. Distances between the OH atom of Y143 or Y181, N atom of C144 or G144, CG2 atom of V191 and either the C7M, C8M, N3 or N5 atoms of FMN in WT or G175D mutant. Distances were calculated on the 800 snapshot structures generated with four independent molecular dynamics simulations using the Bio3D suite in R. Dots represents the average distance ± one standard deviation.
